# QOI8/456: Quality Criteria for Electronic Publications in Medicine

**DOI:** 10.2196/jmir.1.suppl1.e101

**Published:** 1999-09-19

**Authors:** S Schulz, T Auhuber, U Schrader, A Koop, R Kreutz, R Oppermann, H Simm, R Klar

**Affiliations:** ^1^University Hospital, FreiburgGermany

**Keywords:** Online Systems, World Wide Web, Teaching Materials, Clinical Decision Support Systems, Databases

## Abstract

If compared with printed media, the prospect for the success of health-related WWW publications lies in the added value of motivation and efficacy, due to the multi-modal coding of contents, in the possibility to provide search facilities and the flexibility of interaction with the user. An important advantage over the off-line distribution of disks or CD-ROMs is the automated diffusion and update of the contents. Whereas more and more medical WWW sites are launched, their acceptance and utilisation, especially in medical education is still unsatisfactory. Obviously, one reason for this phenomenon is the lack of quality. Many authors, developers and software publishers ignore that the design of high-quality electronic publications is a cost-intensive process. As key qualifications we identify: domain competence, software engineering skills, media production proficiency, GUI design competence, and didactic qualifications. On behalf of the German Association of Medical Informatics, Biometry and Epidemiology (GMDS), the authors have co-ordinated the development of a catalogue of "Quality Criteria for Electronic Publications in Medicine". This catalogue defines as Electronic Publications in Medicine any software containing health-related generic knowledge, with WWW publications being an important group. The criteria catalogue is divided into the sections contents, technical aspects, coding of information, ergonomy, dialogue and didactics. According to these items, typical faults and deficiencies of medical electronic publications are elucidated and possible solutions are given. Our criteria are intended to support the formative evaluation during the development of electronic publications, and to provide a basis for the summative evaluation of medical WWW and offline publications. The catalogue of "Quality Criteria for Electronic Publications in Medicine" is available in German, English, Portuguese and Spanish, at: http://www.imbi.uni-freiburg.de/medinf/gmdsqc/.

